# Influence of Nanoparticles from Waste Materials on Mechanical Properties, Durability and Microstructure of UHPC

**DOI:** 10.3390/ma13204530

**Published:** 2020-10-13

**Authors:** Sahar A. Mostafa, Ahmed S. Faried, Ahmed A. Farghali, Mohamed M. EL-Deeb, Taher A. Tawfik, Stanisław Majer, Mohamed Abd Elrahman

**Affiliations:** 1 Department of Civil Engineering, Faculty of Engineering, Beni-Suef University, Beni-Suef 62511, Egypt; sahar_abdelsalam2010@yahoo.com; 2 Department of Civil Engineering, Faculty of Engineering, Fayoum University, Fayoum 63514, Egypt; asg00@fayoum.edu.eg; 3 Materials Science and Nanotechnology Department, Faculty of Postgraduate Studies for Advanced Sciences (PSAS), Beni-Suef University, Beni-Suef 62511, Egypt; ahmedfarghali74@yahoo.com; 4 Applied Electrochemistry Laboratory, Chemistry Department, Faculty of Science, Beni-Suef University, Beni-suef 62511, Egypt; eldeebm@yahoo.com; 5 Construction and Building Department, Higher Institute of Engineering on 6th of October City, Giza 12592, Egypt; dr.taher_tawfik@csi.edu.eg; 6 Faculty of Civil and Environmental Engineering, West Pomeranian University of Technology, Szczecin, Al. Piastow 50, 70-311 Szczecin, Poland; stanislaw.majer@zut.edu.pl; 7 Structural Engineering Department, Mansoura University, Mansoura 35516, Egypt

**Keywords:** ultra high performance concrete, nanoparticles, waste materials, milling technique, mechanical properties, microstructure, water absorption

## Abstract

This investigation presents the influence of various types of nanoparticles on the performance of ultra high performance concrete (UHPC). Three nanoparticles from waste materials include nano-crushed glass, nano-metakaolin, nano-rice husk ash were prepared using the milling technique. In addition, nano-silica prepared using chemical method at the laboratory is implemented to compare the performance. Several UHPC mixes incorporating different dosages of nanoparticles up to 5% are prepared and tested. Mechanical properties, durability as well as the microstructure of UHPC mixes have been evaluated in order to study the influence of nanoparticles on the hardened characteristics of UHPC. The experimental results showed that early strength is increased by the incorporation of nanomaterials, as compared to the reference UHPC mix. The incorporation of 3% nano-rice husk ash produced the highest compressive strength at 91 day. Microstructural measurements using Scanning Electron Microscopy (SEM), Energy Dispersive X-ray Analysis (EDX), and Thermogravimetric Analysis (TGA) confirm the role of nanomaterials in densifying the microstructure, reducing calcium hydroxide content as well as producing more C-S-H, which improves the strength and reduces the absorption of UHPC. Nanoparticles prepared from waste materials by the milling technique are comparable to chemically prepared nanosilica in improving mechanical properties, refining the microstructure and reducing the absorption of UHPC.

## 1. Introduction

Recently, the ongoing development of construction materials led to improvement in the concrete characteristics and production of new types of concrete with superior properties such as ultra-high performance concrete (UHPC). UHPC characterized by the use of high cementitious materials content, very low water/binder ratio, use of microsilica, superplasticizer and ductile fibers [1]. The optimizing of packing density of concrete constituents results in UHPC with compacted microstructure, which ensures both ultra-high durability and ultra-high mechanical properties [2]. It has many advantages over traditional concrete; higher strength, higher ductility and energy absorption, long service life due to dense microstructure and superior durability, self-healing ability associated with a high amount of unhydrated cement [3]. In some applications special types of concrete are needed to meet the extreme working conditions of high strength and to resist severe uncontrolled conditions [4,5,6,7,8,9]. This is where the new invention of UHPC can be described as a practical and urgent solution. It is the proper material for constructing bridge decks, storage tanks, and shell structures with thin elements [10]. Despite the superior properties of UHPC, their extensive applications are limited due to some drawbacks, including technical problems such as high binder consumption in addition to expensive material cost. It is about 10 times more expensive than traditional concrete, which is attributed to the use of the high amount of cement, high dosage of superplasticizer and expensive steel fibers combined with the need of high quality control. The high cost hinders the wide use of this material. In addition, its production has harmful impacts on the environment compared to conventional concrete [2,11], due to the use of the high content of cementitious materials [12] as well as high temperature curing, which consumes more energy.

With the increasing development of nanotechnology, the influence of nanoparticles on concrete properties has been deeply investigated [1,13]. Different types of nano-based materials are incorporated to improve mechanical properties, durability and to densify the microstructure of UHPC [2]. UHPC is characterized by its perfect distribution of grains through the homogeneous gradient of both coarse and fine particles. In this regard, the incorporation of nanoparticles with an extremely high surface area and small size can improve the packing density of the mixture due to its high filling effect. Consequently, the porosity of UHPC will significantly decrease and the matrix became denser [14,15]. Nanomaterials have very high surface areas compared to other cement-based materials in addition to their nucleation influence, which improve cement hydration efficiently [1,2]. Its addition reduces the porosity, makes the microstructure more dense and homogeneous and improves the bond between fibers and calcium silicate hydrate (C-S-H) [1,16]. Nanoparticles not only act as fillers to reduce porosity and pore diameter, but also as nucleation agents and to refine the interfacial zone between the matrix and aggregate [17,18]. Heikal et al. reported that both the initial and final setting time of the cement are shortened by the addition of 1% nano clay as cement replacement and the one day strength is increased by about 80% [17]. At a later stage, 91 days, the strength increased by about 20%. Nano metakaolin can act as a filler to densify the matrix and transition zone in UHPC [12]. The incorporation of nano-metakaolin is found to be effective in reducing the total porosity and diameter of pores in cement mortar due to its filler effect [1]. However, nano-clay is found to refine the microstructure and reduce chloride migration [12]. Balapour et al. studied the influence of nano-rice husk ash (NRHA), prepared by a ball mill, on the properties of UHPC [19]. The improvement of compressive strength is reported with the addition of NRHA at early and long term ages. Additionally, the chloride migration coefficient is decreased significantly. Similarly, the incorporation of waste glass powder is reported to improve the strength development due to the high content of amorphous silica [20]

From economic aspects, nanomaterials such as nanosilica is expensive, however nanoparticles from waste materials are cheap and do not have a significant cost to concrete [17]. Several techniques can be used to produce nanoparticles such as vaporization at elevated temperatures, precipitation, ball milling and high-speed vertical rotating mill in addition to the chemical synthesis process, which is used to manufacture nanosilica. Ball milling is a popular process to manufacture very fine materials on both a micro and nanoscale. Milling techniques are cost effective with low maintenance cost since it involves less chemical reactions and more mechanical processes. Milling not only breaks bigger particles into nanoparticles, but can also blend different materials and produce new phases [21]. Saleh et al. produced nanosilica with a mean particle size of 50 nm from local silica sand using a ball mill and high temperature treatment [22]. The addition of manufactured nanosilica increases the compressive strength by about 30% at the age of 28 days. Recently, several studies are carried out to investigate the effect of nanoparticles produced from waste materials on the properties of cement composites in order to enhance their mechanical properties and durability [23]. Jabri et al. produced ultra fine nanoparticles from three waste materials; cement kiln dust, copper slag and spent catalyst using the ball milling process [23]. After milling for 4 h, particles with average sizes lower than 100 nm were produced. The addition of nanoparticles makes the transition zone more homogeneous and compacted, as well as enhancing the strength and reducing the capillary porosity [23]. Kutuk-sert used nano colemanite prepared by high-energy ball mill to improve properties of concrete [13]. Norharsi et al. used high energy milling to transfer metakaolin into nano metakaolin with particle size of 176 nanometers and used it in production of UHPC [16]. Incorporation of nano metakaolin decreased the workability of UHPC due to the increased water absorption of metakaolin grains (clay particles). However, compressive strength is improved due to both efficient filler effect and the high pozzolanic reactivity of nano metakaolin.

The main target of this research is to produce UHPC mixture using local available materials incorporating low cost, mechanically prepared nano-waste materials to overcome the problems of the high volume of waste materials. The performances of mechanically prepared nano-waste materials on the properties of UHPC are compared to that of nanosilica, which is the most widely used nanomaterial. Moreover, to reduce the cost of UHPC, a traditional concrete mixer and standard curing method are used to produce and cure the developed UHPC without the need for a special pan mixer or special curing method. In this investigation, three waste materials: metakaolin, crushed glass and rice husk ash, are used as sources to prepare nanoparticles using the milling technique. To compare the effect of these nanoparticles on the performance of UHPC, nanosilica particles prepared using chemical methods is incorporated. To characterize the prepared materials, X-ray diffraction measurements, transmission electron microscopy (TEM) and scanning electron microscopy (SEM) have been implemented. Several UHPC mixes have been prepared and tested in order to examine the influence of different nano-materials on the performance of UHPC. Mechanical properties, durability and microstructure characteristics of different UHPC mixes are measured and evaluated.

## 2. Materials and Methods

### 2.1. Raw Materials

**Figure 1:**
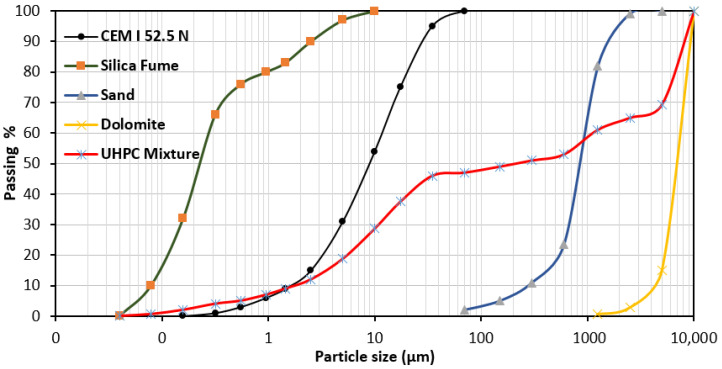
Particle size distribution of cement and silica fume.

Ordinary Portland cement, CEM I 52.5 N provided by Misr Beni Suef company (Beni Suef city, Egypt) according to ASTM C150 is used in this research. The used silica fume is supplied by Sika Egypt Company (Cairo, Egypt). Measured physical properties and chemical composition of the used cement and silica fume are given in Table 1. Figure 1 shows the particle size distributions of cement and silica fume, which was measured by the LISST-Portable|XR (blu) (Sequoia, Bellevue, WA, USA). The coarse aggregate used was crushed dolomite from Ataqa mountain quarry (Suez, Egypt) with maximum nominal size of 6.3 mm with a specific density of 2.65. The used fine aggregate in this research is natural sand with a fineness modulus of 2.94 and specific density of 2.5. Coarse aggregate was used in saturated surface dry conditions (SSD), while fine aggregate was washed and used in dry condition. An additional amount of water equal to the absorption of sand was added to the effective water (w/b). Table 2 presents physical properties of aggregates. Water reducing admixtures were necessary for UHPC due to the low water/binder ratio. Therefore, in this research, a polycarboxylates (PCE) superplasticizer with a density of 1080 kg/m^3^ (trade name Sika Viscocrete 3425 (Sika, Cairo, Egypt)) was used to achieve the appropriate consistency of concrete. To increase the ductility and improve energy the absorption of UHPC, steel fibers were needed. Hooked-end steel fibers with length of 35 mm, aspect ratio of 43.75 and tensile strength of 1100 MPa were used.

### 2.2. Nanomaterials

Different types of nanomaterials were used to evaluate their influences on UHPC properties. In the following section, a short description about the preparation methods of the used nanoparticles is presented.

#### 2.2.1. Nano Silica

In this research work, the used nano silica (NS) was prepared chemically at the faculty of post graduate and advanced studies on Beni Suef, Egypt. The average particle size of the used nano-silica was determined using a Jeol (Tokyo, Japan) transition electron microscope (TEM) and it was 14 nm and the specific surface area was 200 m^2^/kg. X-ray Fluorescence (XRF) was also implemented to measure the chemical composition of different nanomaterials as can be seen in Table 3. NS is chemically prepared by adding hydrochloric acid (HCl) slowly as dropwise to 1000 mL of sodium silicate solution and operating magnetic stirrer until reaching 8–9 PH at room temperature (20–23 °C), thereafter nanosilica was prepared as followes:

- The gel produced remained for 24 h sedimentation of silica at room temperature.
- Filtration stage was important to separate the precipitate from salt water
- Hot distilled water was used for washing several times
- AgNO_3_ solution was used to ensure the efficiency of washing and it’s free from chlorides
- The solution was filtered and the nano silica was dried at 90 °C for about 48 h
- The dried white Nano particles were burnt in high temperature capacity muffle at 600 °C for 2 h; specimens were kept in muffle until cooling to ambient 20 °C.
- Finally, rapid mills for 30 s were done [24].

#### 2.2.2. Nano Waste Materials

Three nanoparticles from different waste materials; nano-rice husk ash (NRHA), nano-waste glass (NWG) and nano-metakaolin (NMK) were prepared by milling as a mechanical grinding process of raw materials until reaching a nano-size. Rice husk ash was prepared by heating at 900 °C for 3 h to obtain a morphs light gray particles with a high percentage of active surface silica. Metakaolin was prepared from dihydroxylation of very fine powder of clay mineral, kaolinite, by burning in a muffle furnace from 20 °C room temperature to 800 °C for 2 h. The high energy milling technique was used for manufacturing the nanoparticles of NRHA, NWG, and NMK from burned rice husk ash, crushed glass and burnt metakaolin, respectively. For the milling process, a ball milling machine was used with two zirconium jars rotating along a horizontal axis with very high speed following the large to small nano-preparation theory, as recommended [25,26,27]. The milling process continued for 4 h for each 50 g using four balls of 20 mm diameter at a speed of 180 rpm.

### 2.3. Raw Materials Characterization

**Figure 2:**
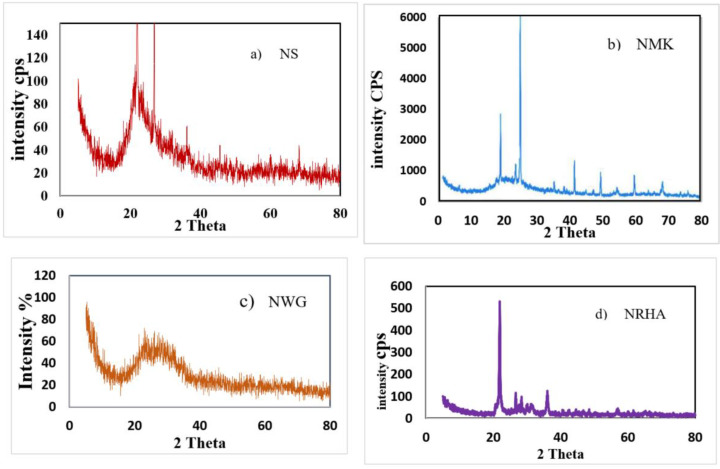
X-ray diffraction pattern of different types of nanoparticles.

**Figure 3:**
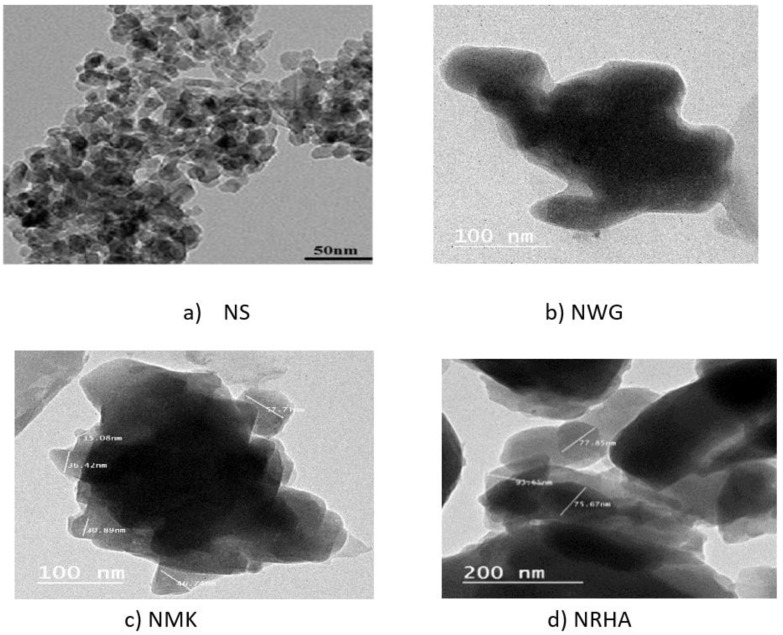
TEM images of different types of nanoparticles.

**Figure 4:**
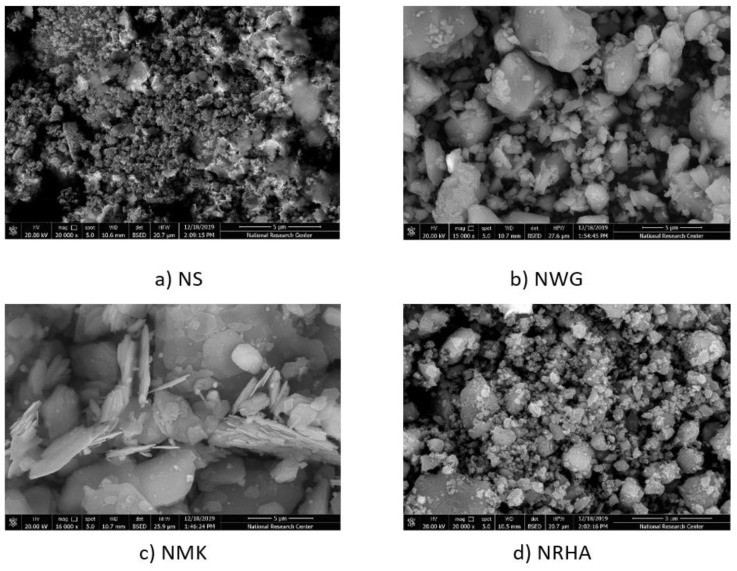
SEM images of different types of nanoparticles.

X-ray diffraction patterns were carried out using the JEOL-2100 electron microscope machine (Jeol, Tokyo, Japan) at the central lab in Beni Suef University, Beni Suef, Egypt, in order to appreciate the amorphous silica for nano-silica (NS), nano-rice husk ash (NRHA) and nano-waste glass (NWG) as well amorphous silica and alumina content for nano-metakaolin (NMK), which predict the pozzolanic efficiency. Figure 2 presents the XRD measurements of NS, NRHA, NWG and NMK. Moreover, TEM and SEM micrographs of nano-materials particles are presented in Figure 3 and Figure 4, respectively. Diffraction graph patterns showed that all four ashes and materials used in this study have high contents of amorphous phases. The low level of crystallinity and one peak at 2 Theta of 22 and 28 in the case of nano-silica observed due to the low unaffected sodium. The average particle size for nano-waste materials was 50 nm, 30 nm, and 50 nm for NWG, NMK and NRHA, respectively. Figure 4 shows the morphs state of nano-materials due to non-homogeneity scale and shape of particles, but the NMK graph explains some crystalline of hexagonal shape. As can be obviously seen from the TEM images, most nanoparticles have irregular shapes. The XRF analysis results of nano-waste materials are presented in Table 3.

### 2.4. Concrete Mix Design

The main target of this investigation was to study the influence of nanoparticles from different sources and prepared by different techniques on the properties of UHPC. To achieve this target, 15 UHPC mixes have been prepared and tested. The reference mixture (coded C0) of UHPC was selected after being developed from a previous study [16]. NS, NWG, NMK and NRHA were added with different dosages ranged from 1% to 3% by the mass of the binder content in the first three nano-materials and ranged from 1% to 5% in the case of the last type. Table 4 presents the proportions of concrete mixtures. In all mixes, cement content was 900 kg/m^3^, where the silica fume content was about 13% by the mass of the binder content. The water added to the binder ratio was kept at 0.18 and the water in the NS solution and superplasticizer were deducted from the added water. The superplasticizer dose was 2.17% of the mass of the binder and kept constant for all mixtures. Steel fibers were added as 1% of the volume of concrete. All mixes were similar and the only change was the nanomaterial type and dosage.

### 2.5. Mixing Procedure

The mixing method of UHPC had a significant influence on its fresh and hardened properties. The influence of various parameters such as mixing time, speed and sequence of adding the ingredients of concrete into the mixer have been studied by several researchers and proved to have a significant influence on the produced material [28,29,30]. Therefore, in this investigation, all concrete mixes have been prepared with the same mixing method and procedure. Five Specific mixing steps were followed in sequence to get UHPC, so the water/binder ratio was minimized. The following steps explain the procedure during mixing:

A magnetic stirrer was used to ensure the full dispersion of nano-materials (NS, NWG, NMK and NRHA) with 50% mixing water and a hyper plasticizer

- Fine and coarse aggregate were mixed together for 1 min, thereafter, cement and silica fume were incorporated and dry mixed for 2 min
- 50% of estimated mixing water was added during mixing and continued for 6 min
- The nano materials’ solution with superplasticizer and water were added and mixed for 5 min

Finally, the fibers were then added to the mixture and the concrete mixer was run for an additional 6 min. Samples were casted and kept in a room temperature at 20 °C and relative humidity (RH) not less than 95% for approximately one day. The samples were demolded and the curing process took place at the laboratory temperature (21 ± 2 °C) in a water basin till the testing age.

### 2.6. Experimental Tests

#### 2.6.1. Mechanical Properties

The compressive strength test for all UHPC mixtures were carried out according to ASTM C39 on cubic samples with an edge length of 150 mm [31] using a 3000 KN capacity digital compressive testing machine. The tests were performed to measure the strength of concrete at different ages of 7, 28 and 91 days. The mean values of three tested samples of each mix was considered and presented in this research. The splitting tensile strength was conducted according to ASTM C 494/C495M [32] on cylindrical samples of 100 × 200 mm at the age of 28 days. Three samples were tested and the average value was considered. For flexural strength determination, concrete beams with dimensions of 10 × 10 × 700 mm were tested according to ASTM C1609/C1609 M [33].

#### 2.6.2. Durability

The sorptivity of concrete was measured according to ASTM C1585 by measuring the rate of absorbed water by concrete [34] as an indicator for durability. The test was performed at 28 days. Cylindrical concrete samples were dried and kept for 24 h with silica gel, which had the ability to absorb moisture and keep the specimens dry. The initial dried mass of the cylinders was first measured with a high accuracy balance after coating the perimeter to permit the flow of moisture in one direction only. Thereafter, the samples were partially immersed in water so that only 3–5 mm of the bottom of the sample was submerged under water. Then, the increase of the sample mass was determined repeatedly according to the time intervals mentioned in ASTM C1585 [35]. The sorption coefficient (S) can be calculated as the slope of the best fit line between the mass increase and the square root of time (t), as given in Equation (1):

(1) $$S=\frac{I}{\sqrt{t}}$$

#### 2.6.3. Chemical and Microstructural Characteristics

For estimating the cement hydration products in the case of adding different nanomaterials, thermogravimetric analysis was examined by specimens exposed to different temperatures under an Argon specified atmosphere [36]. To carry out the measurements, cement pastes were prepared from nanoparticles of NS, NWG, NMK, and NRHA and cement and kept until 28 days. Then, the hardened pastes were milled to fine powder. Then, the specimens were put in the device for heating operation starting from ambient temperature, ending at a severe argon atmosphere up to 1000 °C for 98 min with a step of 10 °C/min. The following formula was used to assess the calcium hydroxide (CH) content, which was the second cement hydration product after C-S-H from the TGA curve:

(2) $$\mathrm{CH}\;(\%)\;=\;{\mathrm{WL}}_{\left(\mathrm{CH}\right)}\;\times \;\frac{{\mathrm{MW}}_{\left(\mathrm{CH}\right)}}{{\mathrm{MW}}_{\;\left(H_{2}O\right)}}$$

where WL_(CH)_, MW_(CH)_ and MW_(H_2_O)_ symbolized as the first one as the mass loss during the dehydration of CH in the temperature ranged from 400–500 °C, Ca(OH)_2_ molecular weight (CH component) and finally is the water molecular weight, respectively.

Five concrete samples including the reference mix and 4 other specimens with 1% NS, 1% NWG, 1% NMK and 3% NRHA are selected based on the compressive strength results. Small sample from each concrete mix was crushed into small pieces and milled to powder. The powder X-ray diffraction method was used for the analysis and explained the characteristics of concrete microstructure [35]. The XRD analysis was done at the X-ray diffractometer in the central lab in the faculty of post graduate for advanced studies (PSAS), Beni Suef, Egypt. The tube voltage and X-ray current are constant at 40 KV and 40 mA, respectively. Raw Data Origin XRD measurement (*.XRDML) and Minimum step-sized Omega equal 0.0001 and scan step-size equaled 0.0130° with flat sample stage holders and EMPYREAN Diffractometer system (Malvern, Germany). The data was measured by the PANalytical (version 4.8, x’pert Plus).

Micro-structural analysis was carried out on thin polished sections and small fragments of dimensions (10 × 10 × 10) mm from the core of the concrete cubes. Samples from reference mix and other mixes with different nanomaterials are examined. The observation was performed with SEM model Philips XL 30 attached with an EDX unit (SEM-Tech, Worcester, MA, USA), with a high voltage of 20.00 KV, magnification 10× *g* up to 400,000× *g* and resolution for W (3.5 nm).

## 3. Results and Discussion

### 3.1. Mechanical Properties

#### 3.1.1. Compressive Strength

**Figure 5:**
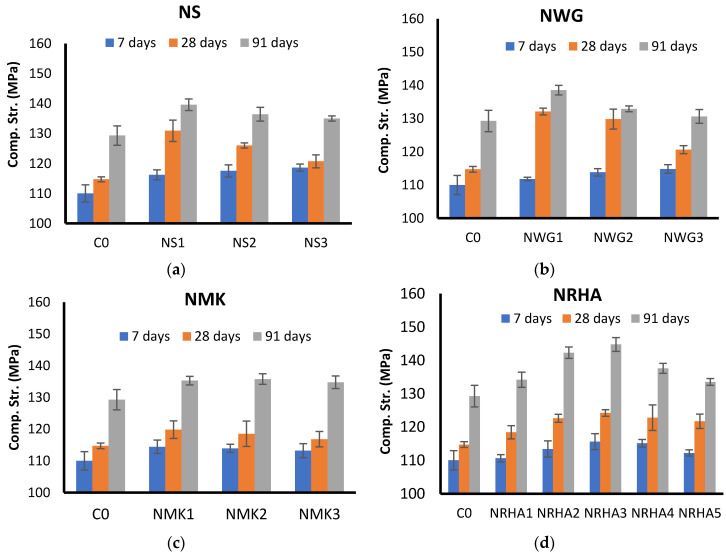
Influence of different nanomaterials on compressive strength of UHPC at 7, 28, and 91 days: (**a**) Nanosilica, (**b**) Nano waste glass, (**c**) Nano metakaolin, (**d**) Nano rice husk ash.

Figure 5 shows the experimental results of all UHPC mixes at the ages of 7, 28 and 91 days. It is clear from Figure 5a that the incorporation of nanosilica improves the compressive strength of UHPC at all ages in comparison to the control mix (C0). The early strength, at the age of 7 days, was significantly improved due to the addition of nanosilica. This can be attributed to both physical and chemical roles of nanosilica. It works as a very fine filler that fills the pores and densifies the microstructure. However, it can chemically react with CH in the pozzolanic reaction to produce additional C-S-H, which improves the strength [37,38]. At 28 days, the increase in compressive strength is still obvious; however, at a later age (91 days), the increase in compressive strength was reduced. The experimental results reflect that 1% addition of nanosilica is more effective than other doses in increasing the strength. Similar results have been noticed by Heikl et al. [17], where 20% improvement in strength at the age of 91 days with the incorporation of 1% nano-clay.

However, the incorporation of NWG has a similar influence on UHPC strength as NS at 7 and 28 days; however, at 91 days, the influence vanished and depends on the dosage. It is clear from Figure 5b that a dosage of 1% of NWG is more effective than other dosages at all ages. This can be attributed to the possibility of agglomeration and non-effective dispersion when a high dosage of NWG is used. With high agglomeration, the specific surface area of the nanoparticles is reduced and its pozzolanic reactivity is decreased, leading to a retarding of the cement hydration. As a result, the strength improvement associated with the incorporation of nanomaterials is diminish and a less dense microstructure is formed. Moreover, due to the agglomeration of nanoparaticle, there was a creation of so-called “weak-zones”, resulting in the formation of crack or local decrement of the mechanical strength of microstructure

Figure 5c presents the experimental results of the compressive strength of UHPC with NMK. A very marginal improvement in strength can be observed in all ages associated with the incorporation of nano metakaolin. At early ages, the influence of NMK in accelerating the hydration and improvement of early strength is very weak. This influence may be due to the chemical composition and physical characteristics of NMK. This finding was also reported by Reference [39], and was attributed to the inefficient filling ability of NMK in highly compacted UHPC mixtures. The addition of 1% NMK appears to be more efficient in improving the strength at 28 days where it reached about 120 MPa compared to other dosages of 2% and 3%, which exhibit a compressive strength of 118 and 116, respectively. At 91 days, the difference between the strength of NMK1 and the control mix is minimized to about 1% only. Higher dosages of NMK do not have significant influences of compressive strength at all ages.

The results of the mixes with NRHA are presented in Figure 5d. In the case of nano-rice husk ash five dosages have been tested and compared to the reference mix; 1, 2, 3, 4, and 5%. At early ages, it is obvious from the experimental results that small increases in early strength are observed for mixes with NRHA. However, at later ages, 28 and 91 days, the improvement in compressive strength is more pronounced. It is clear that, as the dosage on NRHA increases the compressive strength increase up to 3% dosage, it exhibited the highest compressive strength. These results are in good agreement with Balapour et al. study, who reported an improvement of compressive strength at both early and later ages [19]. With increasing dosages above 3%, the strength start to decrease, as in the cases of 4% and 5%, but still higher than the reference mix. Due to its very small particles and the large specific surface area, nanoparticles tend to agglomerate when a high dosage is used. This agglomeration of particles makes the microstructure less homogeneous with a less uniform distribution of pores and C-S-H within the pore solution and consequently the compressive strength does not improve significantly.

**Figure 6:**
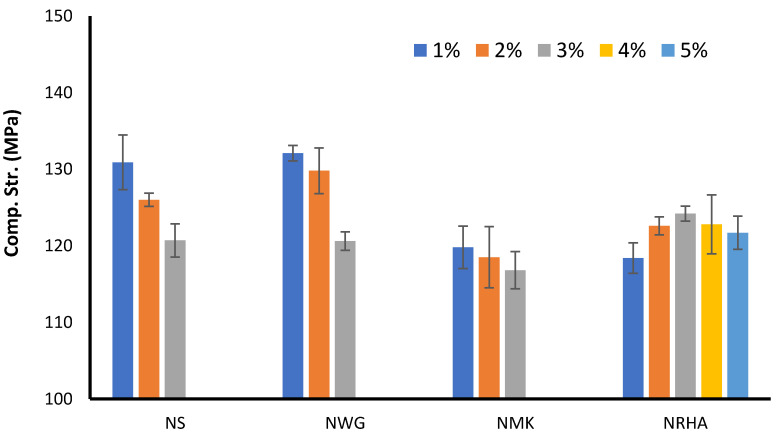
Comparing the influence of different nanomaterials on UHPC strength at 28 days.

Figure 6 presents a comparison between the effects of various types of nanomaterials on the strength development of UHPC at 28 days. The reference mix exhibited a compressive strength of about 115 MPa after curing at normal conditions without heating. The addition of nanoparticles improves the strength depending on their type and dosages. The performance of NS and NWG is very similar. Both materials increase the strength to about 132 MPa when 1% is added to the mixture. The compressive strength increased by about 13%. This is due to the important role of very fine nanoparticles in densifying the pore structure (filler effect) as well as their pozzolanic reaction with CH to form more C-S-H. Moreover, the nanoparticles have the ability to work as seeding agent to improve the strength development. However, with increasing dosages of the strength, development decreases to about 120 MPa when 3% is added. The reduction of compressive strength in both cases with higher dosage could be due to the agglomeration and clumping of the particles, which results from the nonhomogeneous distribution of the particles. This behavior reduces the role of nanoparticles in accelerating the strength development. Compared to NMK, it is obvious from the experimental results that NMK is not so efficient as NS or NWG in improving the strength, though the strength is still higher than that of the reference mixture. This can be attributed to the high pozzolanic reactivity of nanosilica and nano-waste glass due to the high content of amorphous silica compared to nano-metakaolin, which is a clay mineral. Similar to the NS and NWG, the strength is gradually decreased with increasing dosages of NMK above 1%. For nano-rice husk ash mixtures, the behavior is a slightly different from that of other nanoparticles; the strength is increasing with increasing the dosage of NRHA until 3%. However, with higher dosages, 4 and 5%, the strength starts to decrease due to the increasing amount of added nanoparticles more than the saturation level. By comparing the experimental results at all ages. It can be detected that NS and NWG have a significant influence on the early strength while the role of NMK and NRHA is very marginal.

In contrast, at later ages (91 days) the strength of NRHA mixes is the highest and it reached about 145 MPa, which is about 9% higher than the reference mix, as presented in Figure 5. The reason for the difference performance of the examined nanoparticles can be attributed to the difference in their chemical composition and particle shapes. The optimum dosages for the used nanoparticles are different from one material to another depending on its chemical, physical and mineralogical characteristics. From the presented results and previous discussion it can be concluded that some types of nano-waste materials, which were prepared with the milling process, such as NWG, NMK and NRHA are comparable to chemically manufactured nanoparticles such as NS. This reflects the possibility of using low cost sustainable nanoparticles in producing UHPC without altering its compressive strength negatively.

#### 3.1.2. Flexural and Tensile Strength

**Figure 7:**
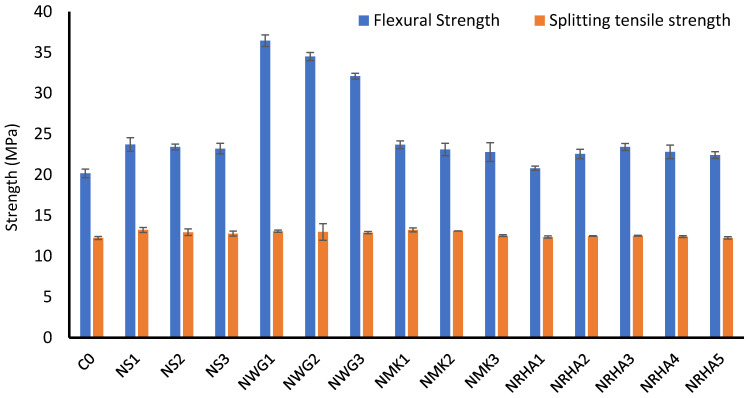
Flexural and tensile strength of UHPC at 28 days.

The flexural and tensile strength tests have been performed on concrete specimens at 28 days and the results can be found in Figure 7. It is clear from the results that the incorporation of nanoparticles improved the flexural and tensile strength of all mixes in comparison to the reference mix. The addition of nanoparticles densifies the microstructure and refines the transition zone between the cement matrix and aggregates. In addition, due to its high pozzolanic reactivity and filler effect, it reduces the volume of weak calcium hydroxide and replaces it with C-S-H, which has a higher strength. NWG appears to have the best influence on flexural strength development when 1% is added. With more dosages, the strength tends to decrease. This performance is similar to the compressive strength results of the NWG mixes. For splitting tensile strength, the difference between the results is small, but none of the mixes exhibited tensile strength smaller than the reference mix. In addition, compared to NS, the performance of NRHA is similar, while the influence of NWG is more pronounced.

### 3.2. The Effect of Nano Materials Contents on Durability Properties of UHPC

**Figure 8:**
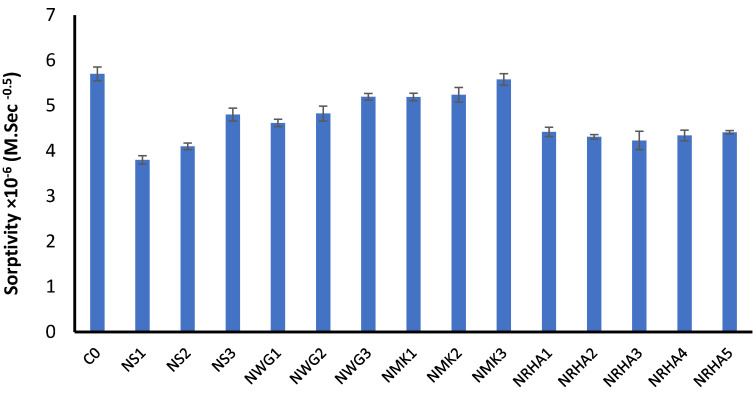
Experimental results of sorptivity of different concrete mixes at 28 days.

**Figure 9:**
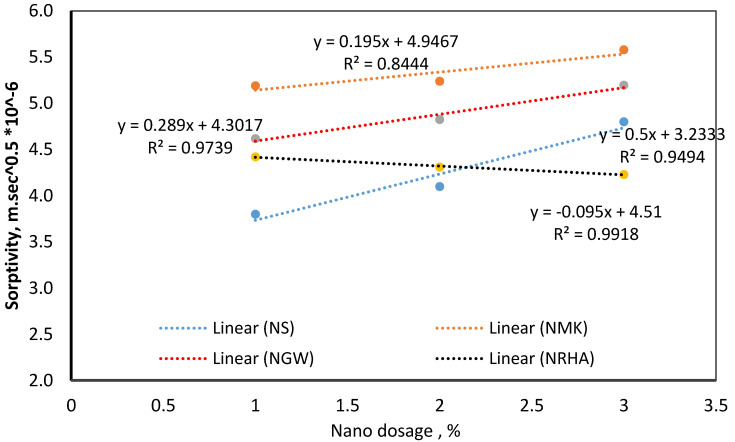
Accumulative sorptivity results of various UHPC specimens at 28 days of curing.

In this investigation, water sorptivity is evaluated as an indicator for the durability of concrete. In the test method, a concrete sample is partially immersed in water within a container. The main force that absorbs the water inside the concrete sample is the capillary suction, which depends mainly on the number and continuity of capillary pores. The mass increase in every sample is recorded with time and the sorptivity coefficient can be calculated, as presented in Figure 8. It is obvious from the experimental results that all concrete mixes have sorptivity lower than the reference mix. The addition of nanoparticles fills the pores due to its filler effect. Moreover, its high pozzolanic reactivity refines the pore system and reduces the effective capillary pores, which govern the water absorption. For NS and NWG mixes, addition of 1% is more effective in decreasing the sorptivity of UHPC mixes. However, with increasing the dosage to 3%, the capillary suction is increased. Figure 9 shows the influence of different dosages of nanoparticles on the sorptivity of UHPC mixes. It is clear that, for most nanoparticles, increasing the dosages above 1% exhibited an increase in its sorptivity. This results agree well with the results of mechanical properties where the compressive strength start to decrease with increasing the nanoparticles dosage up to 3% due to the possibility of agglomeration of very fine particles leading to an increase in capillary porosity and the production of more porous microstructure compared to 1% dosage. The same trend can be detected for NMK. A similar conclusion has been reported by Wu et al. who stated that the incorporation of nano-metakaolin is very effective in reducing the total porosity and the diameter of pores sue to its filler effect [1]. For NRHA, the influence of its addition on sorption is significant. The optimum dosage of NRHA that gives the lowest value of sorption is 3%. It is also obvious from the figure that nanosilica and nano-rice husk ash have the lowest sorption values. Their role in refining the microstructure and reducing the capillary porosity is more obvious than other types of nanoparticles. This can be refer to the pozzolanic reaction and to their high efficiency as filler and seeding agents compared to other types.

### 3.3. Hydration and Microstructural Characteristics

#### 3.3.1. Thermogravimetric Analysis

**Figure 10:**
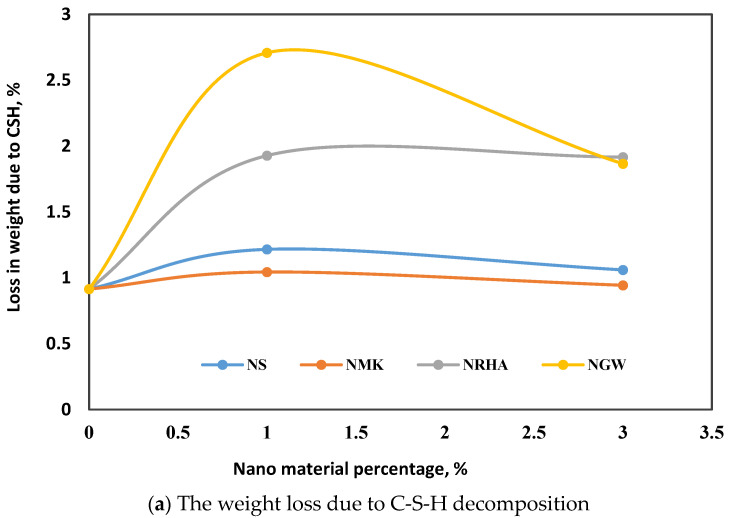

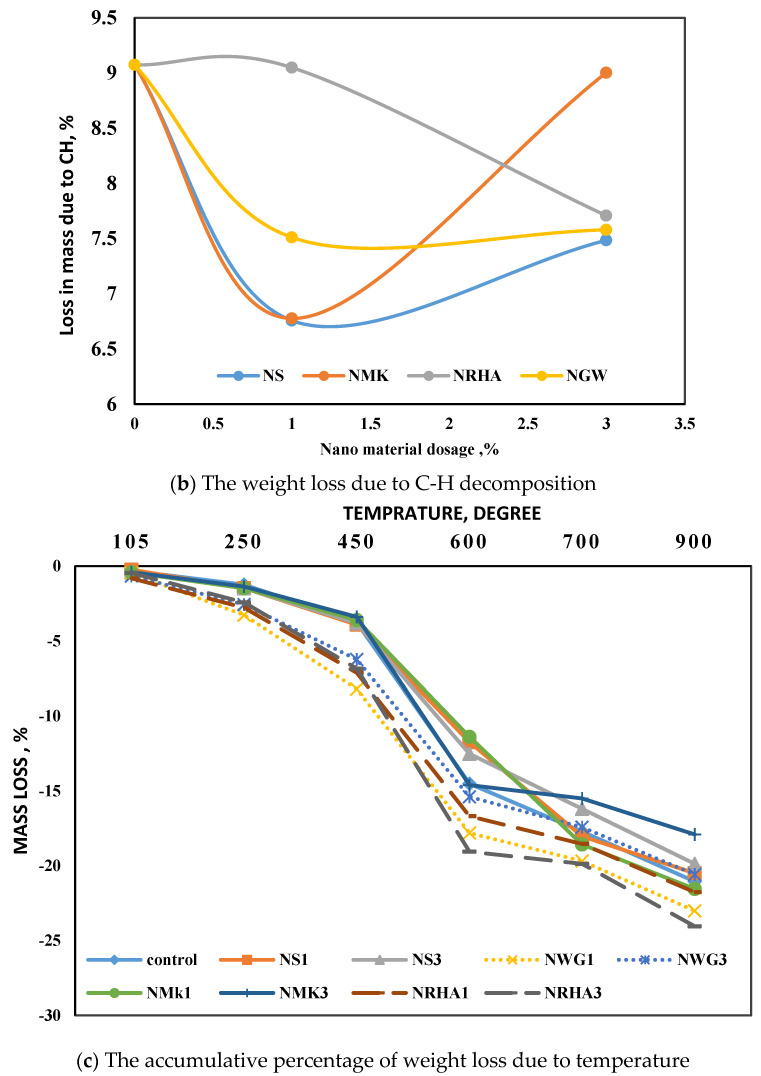
TGA for UHPC mix.

A thermogravimetric analysis has been implemented to evaluate the effect of different nanoparticles on the phase’s formation of UHPC pore solution at the age of 28 days. In this investigation, C-S-H is considered to decompose at the temperature of 100–250 °C, while the drop in the figure at the temperature 400–500 is due to the decomposition of CH. Up to 250 °C, in addition to the decomposition of C-S-H, ettrinigite also is one of the hydration products as reflected by XRD, which can be decomposed at similar temperature ranges. However, C-S-H is the most prominent and appeared at different positions with higher density. It is clear from Figure 10a that incorporation of nanoparticles increases the content of C-S-H. In the case of 1% NS, the amount of C-S-H is more than the reference mix. However, with increasing the dosage up to 3%, it tends to decrease. Compared to NS, both NWG and NRHA appear to be more efficient in accelerating the hydration of cement and increasing the pozzolanic reaction with CH. The highest content of C-S-H was found in the case of nano-waste glass with 1%. By increasing the dosage, the amount of the formed C-S-H is decreased. In the case of NRHA, the content of C-S-H is increased at 1% and is approximately constant with higher dosages. These findings can be confirmed from the results of the mass loss due to the decomposition of CH at a temperature of 400–500 °C, as shown in Figure 10b. It is clear that the addition of 1% of nanoparticles led to a decrease in CH content in the case of NS, NGW, and NRHA. The decrease in CH content is attributed to the pozzolanic reaction with nanomaterials, which consume the CH (Figure 10b) and create additional C-S-H (Figure 10a). The increase in the weight loss in Figure 10a means the increase in the formed C-S-H due to the pozzolanic reaction between calcium hydroxide and nanoparticles. For NRHA, the lowest C content was found with the addition of 3%. That mean high content of C-S-H is produced at that dosage. In cement composites, calcium silicate hydrates (C-S-H) is the component responsible for the binding capacity of concrete, while CH is the secondary product of cement hydration and its contribution in strength is very limited. In other words, mixtures with low CH and high C-S-H have higher strength and vice versa. Figure 10c presents TGA curves for the control mix as well as other mixes with different nanoparticles. The mass loss above 550 can be due to the dehydration of C-S-H and carbonation. The experimental results of compressive strength and the outputs of TGA measurements confirmed this claim. With the incorporation of nanoparticles, cement hydration accelerated, pozzolanic reaction takes place, CH is consumed and more C-S-H is formed. As a consequence, strength and mechanical properties are improved. In addition, with formation of C-S-H, the pore solution becomes denser and the continuity of capillary pore pats are interrupted [40,41], leading to a strong reduction in concrete sorptivity, as observed from the experimental results.

#### 3.3.2. X-ray Diffraction (XRD)

**Figure 11:**
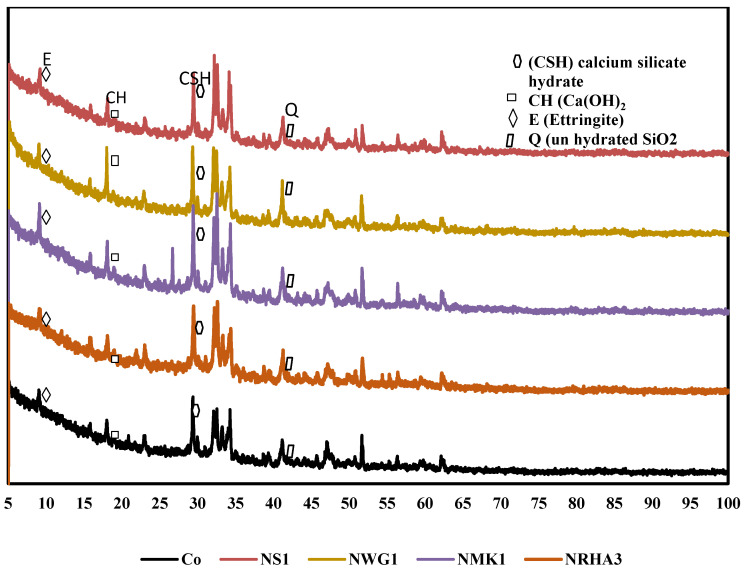
XRD for cement pastes with different nanomaterials.

X-ray Diffraction has been performed on four hardened UHPC specimens containing nano-particles with corresponding optimum compressive strength results at (1%NS, 1% NGW, 1% NMK and 3% NRHA), compared to reference paste without nano-particles. Figure 11 shows the XRD pattern at 28 days. The main phases induced in UHPC were observed at the correct position; crystalized C-S-H (at 29.6°), Ca(OH)_2_ (at 18.1°). The peak at 26.1°, 26.8°, 39.6°, 42.5°, 50.3° and 60.0° is due to the presence of SiO_2_ unhydrated cement clinker (C_2_S/C_3_S, at 32.3° and 32.8°), and ettrinigite (at 9.6°). The intensity of the peak in all the phases is proportional to the amount of formed products. The increase in intensity refers to the high amount of the product. There is a significant difference in peaks between CO and other specimens with nano-particles (NS1, NWG1, NMK1 and NRHA3) at 29.6°. The recorded heights are (436, 607, 642, 952.7 and 687 Cts) in the case of different hydrated pastes (C0, NS1, NWG1, NMK1, and NRHA3) respectively. Therefore, it can be concluded that nano-particles with high silica content produce denser C-S-H. The secondary hydration product peaks are compared at the same position of 18.1° and the intensity was revealed as (15.64%, 8.7%, 13.3%, 10.2% and 9.44%) for (CO, NS1, NWG1, NMK1 and NRHA3). The less intensity of C-H resulted in the case of incorporating nano-particles ensure its vital role in consuming the residual content in C-S-H formation. At 9.6°, low intensity of ettrinigite composed in the case of nano-particles.

#### 3.3.3. Scanning Electron Microscope

**Figure 12:**
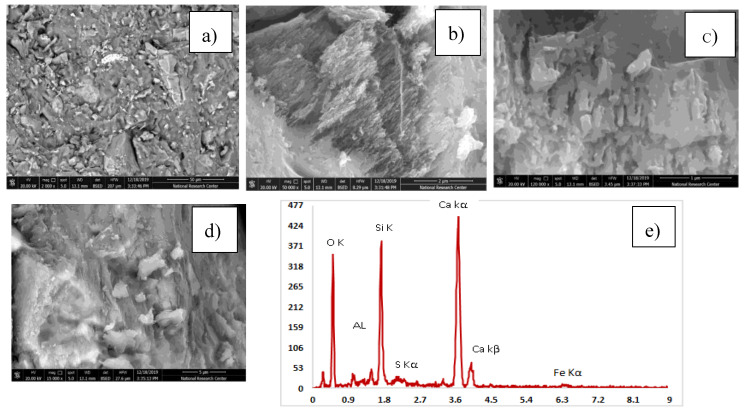
(**a**–**d**) SEM images at different magnification factor and (**e**) EDX EDX for the control mix of UHPC.

**Figure 13:**
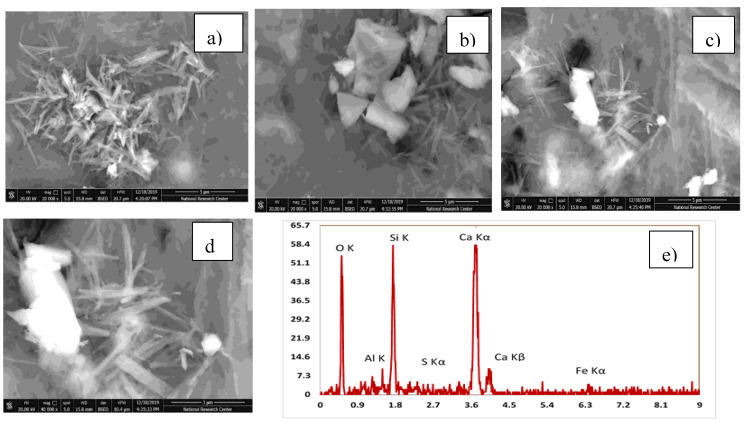
SEM and (**a**–**d**) SEM images at different magnification factor and (**e**) EDX for the UPC mix incorporating 1% nanosilica.

**Figure 14:**
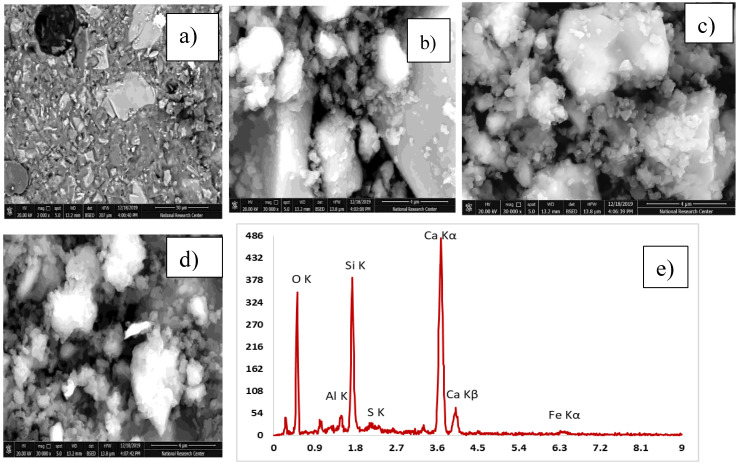
SEM and (**a**–**d**) SEM images at different magnification factor and (**e**) EDX for the UHPC mix incorporating 1% nano-waste glass.

**Figure 15:**
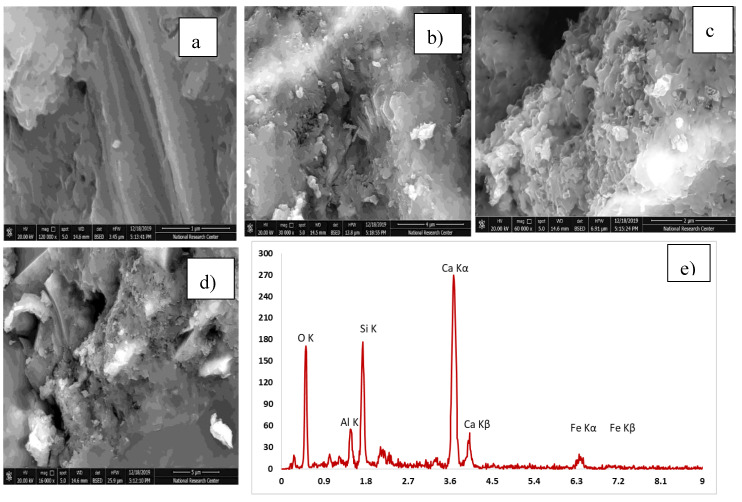
SEM and (**a**–**d**) SEM images at different magnification factor and (**e**) EDX for the UHPC mix incorporating 1% nano-metakaolin.

**Figure 16:**
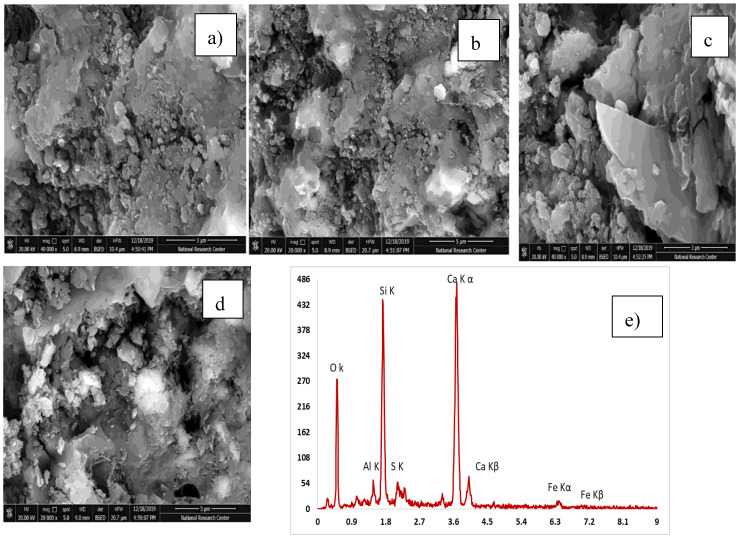
SEM and (**a**–**d**) SEM images at different magnification factor and (**e**) EDX for the UHPC mix incorporating 3% nano-rice husk ash.

Table 5 explains the EDX results for four mixes with the optimum compressive strength compared to the control mix. The analysis of the results has been compared. High pozzolanic reactions occurred. Table 6 shows the Ca/Si ratio, which is known as the indicator for C-S-H gel. EDX explains a high percentage of Ca/Si of 2.13 in the C-S-H gel. Figure 12, Figure 13, Figure 14, Figure 15 and Figure 16 explain the SEM micrographs of five UHPC mixes. Dense sections and low micro cracks was observed in the control mix and dense sections was also represented due to the continuity of the hydration process in the case of a high amount of fine and ultra fine proportions in UHPC compared to traditional concrete. Reinforcing UHPC with nano-particles with a high percentage of silica was the main reason for forming homogenous sections, obtaining dense and compacted sections, decreasing the size of calcium hydroxide crystals and producing new calcium silicate hydrate responsible for improving strength. Finally, tight bond morphology without apparent micro-cracks were observed and significant ITZ in UHPC with nano-particles due to its efficiency in the hydration process.

SEM micrographs are examined for the reference of UHPC mix CO and four samples are taken from UHPC with the optimum strength reinforced with different nano-particles, as shown in Figure 12, Figure 13, Figure 14, Figure 15 and Figure 16). All mixes showed a dense microstructure compared to the well-known microstructure of conventional and developed high strength concrete. Tight bond and small thickness of micro cracks were observed in the vicinity of aggregate ITZ only in UHPC sample, but no micro-crack propagations were observed in all mixes incorporating nano-materials. The low water/binder ratio plays vital role in restricting the voids over the spread as the high mount of fine materials can increase the viscosity of cementitious materials and decrease the air entrapped ability at the same time as the potential implications fine particles like silica fume and ultra-fine particles (nano-materials) are contributing to the pozzolanic reaction environment and developing the gel produced by forming homogenous sections with low calcium hydroxide crystals and lowering the hardened concrete porosity. On pointing to the EDX analysis, the Ca/Si content is the favorable indication for the pozzolanic reaction. The results showed that the Ca/Si mole ratio equaled 2.13 in UHPC, this high percentage is due to the superior properties of this type of concrete and this value is enhanced and recorded (2.53, 2.72, 3.5, and 2.31) in the case of NS, NWG, NMK, and NRHA, respectively. The results ensured the activity of nano-materials especially amorphous silica saturated types in enhancing the microstructure of UHPC. Moreover, their addition contributes significantly to the formation of a dense microstructure with small pore sizes due to the improved arrangement and packing of the particles with small sizes [42].

## 4. Conclusions

This investigation studied the influence of different nanoparticles prepared from waste materials on the properties of UHPC. The concluding remarks can be summarized as follows:

- The compressive strength is significantly improved by adding the nano-particles. Mixes incorporating nano-silica, nano-waste glass and nano-Metakaolin achieved the maximum strength at 1% NS, 1% NWG and 1% NMK due to their compactness, whereas, nano-rice husk ash increases the compressive strength, especially at later ages due to its characteristics of saving water at early ages and promoting them to share in the hydration process, with the optimum strength obtained at 3%. Meanwhile, the maximum compressive strength was achieved at 3% nano-rice husk ash.
- All four types of nano-materials used increased the splitting and flexure strength of concrete.
- The highest flexure strength was achieved at 1% of Nano Waste Glass of 80% comparing to 17%, 17%, 15% in case of NS1, NMK1 and NRHA3, while the highest splitting strength was achieved at 1% nano-silica and 1% nano-metakaolin of 9% whereas nano-waste glass and nano-rice husk ash achieved a 4% and 7% increase.
- Nano waste glass was found to be the most suitable admixture to be used in UHPC due to its lowest production costs as well as low dosage required to obtain the most superior properties among tested UHPCs
- Adding nano-particles reduced the value of absorption according to sorptivity test. The effect was more pronounced by adding 1%NS as it reduced the water absorbed ratio with 50%.
- Compared to control mix, introducing nano materials in concrete mix led to increasing C-S-H and decreasing C-H.
- SEM analysis showed compact, thick sections with no porosity.
- EDX analysis pronounced that adding nano particles increased Ca/Si ratio, hence accelerating the internal reactions. The maximum percentage achieved at 1% nano-metakaolin and nano-waste glass of 34% and 26% compared to control mix, nano-silica, and nano-rice husk ash achieved an increase of 17% and 8.4%, respectively.
- As the outcome of this research, UHPC can be produced without the need for high cost techniques such special mixer or the special curing method. Moreover, it can be optimized using mechanically prepared nanoparticles from waste materials, which is less expensive than other types of nanomaterials available in the market.

## Figures and Tables

**Table 1:** Physical properties and chemical composition used fine materials.

Material	CaO	SiO_2_	Al_2_O_3_	Fe_2_O_3_	MgO	Na_2_O	K_2_O	SO_3_	LOI	Density (g/cm^3^)	Surface Area (cm^2^/g)
CEM I 52.5 N	63.4	21.2	5.5	3.21	0.7	0.1	0.5	2.4	2.3	3.15	3500
Silica fume	0.2	97	0.1	1.0	0.15	0.10	0.2	0.1	2.2	2.15	

**Table 2:** Physical properties of the used aggregates.

Physical Properties	Crushing (%)	Absorption (%)	Clay and Fine Materials%	Bulk Density (kg/m^3^)	Specific Gravity (g/m^3^)
Fine aggregate	-	1	1	1700	2.5
Coarse aggregate	7	5.0	0.5	1720	2.65

**Table 3:** Chemical composition of NS, NWG, NRHA, and NMK (wt.%).

Items	SiO_2_	Al_2_O_3_	Fe_2_O_3_	CaO	MgO	SO_3_	K_2_O	Na_2_O	Cl	TiO_2_
NS	95.39	0.15	1.11	0.43	0.09	0.05	0.030	1.790	0.71	-
NWG	72.58	0.17	1.11	12.12	2.09	0.19	0.030	11.700	0.01	-
NMK	89.6	0.9	2.0	0.43	2.0	-	4.55	-	-	0.7
NRHA	73.05	1.16	0.18	3.50	1.45	0.47	5.670	2.740	1.81	-

**Table 4:** Mixture proportions of UHPC.

No.	Cement	SF	Fine Aggregate	Coarse Aggregate	Water	SP	Fiber (V%)	NS %	NWG %	NMK %	NRHA %
	kg/m^3^										
CO	900	135	349	776	186.3	22.5	1	-	-	-	-
NS1	900	135	349	776	186.3	22.5	1	1	-	-	-
NS2	900	135	349	776	186.3	22.5	1	2	-	-	-
NS3	900	135	349	776	186.3	22.5	1	3	-	-	-
NWG1	900	135	349	776	186.3	22.5	1	-	1	-	-
NWG2	900	135	349	776	186.3	22.5	1	-	2	-	-
NWG3	900	135	349	776	186.3	22.5	1	-	3	-	-
NMK1	900	135	349	776	186.3	22.5	1	-	-	1	-
NMK2	900	135	349	776	186.3	22.5	1	-	-	2	-
NMK2	900	135	349	776	186.3	22.5	1	-	-	3	-
NRHA1	900	135	349	776	186.3	22.5	1	-	-	-	1
NRHA2	900	135	349	776	186.3	22.5	1	-	-	-	2
NRHA3	900	135	349	776	186.3	22.5	1	-	-	-	3
NRHA4	900	135	349	776	186.3	22.5	1	-	-	-	4
NRHA5	900	135	349	776	186.3	22.5	1	-	-	-	5

**Table 5:** EDX results in different UHPC mixes with and without nanoparticles.

Element	Control (wt.%)	NWG (wt.%)	NS (wt.%)	NRHA (wt.%)	NMK (wt.%)
C K	4.21	7.48	5.94	4.68	6.7
O K	53.2	50.64	51.46	45.89	49.29
AlK	1.93	0.95	1.69	1.34	2.45
SiK	11.44	10.23	10.86	12.65	8.29
CaK	24.42	27.85	27.56	29.26	29.04
FeK	1.29	0.87	2.49	2.12	4.23
NaK	2.59	1.98	100		
MgK	0.93				
S K				3.27	
K K				0.79	

**Table 6:** Ca/Si ratio of different concrete mixes.

Mix ID	CO	NS1	NWG1	NMK	NRHA3
Ca/Si	2.13	2.53	2.72	3.5	2.31
